# High throughput *in situ* imaging reveals widely occurring diel vertical migration among phytoplankton

**DOI:** 10.1093/ismeco/ycag049

**Published:** 2026-03-09

**Authors:** Karin Garefelt, Bengt Karlson, Michael L Brosnahan, Kaisa Kraft, Anders Torstensson, Jukka Seppälä, Allan Cembella, Anders F Andersson

**Affiliations:** Department of Gene Technology, Science for Life Laboratory, KTH Royal Institute of Technology, 171 65 Solna, Sweden; Research and Development, Oceanography, Swedish Meteorological and Hydrological Institute, 426 71 Västra Frölunda, Sweden; Biology Department, Woods Hole Oceanographic Institution, Woods Hole, MA 02543, United States; Research Infrastructure Unit, Finnish Environment Institute, 00790 Helsinki, Finland; Community Planning Services, Oceanography, Swedish Meteorological and Hydrological Institute, 426 71 Västra Frölunda, Sweden; Research Infrastructure Unit, Finnish Environment Institute, 00790 Helsinki, Finland; Helmholtz Centre for Polar and Marine Research, Alfred Wegener Institute, 27570 Bremerhaven, Germany; Department of Gene Technology, Science for Life Laboratory, KTH Royal Institute of Technology, 171 65 Solna, Sweden

**Keywords:** phytoplankton, diel vertical migration, imaging flow cytometry, imaging FlowCytobot (IFCB), microbial ecology, Baltic Sea

## Abstract

Phytoplankton undertake daily vertical migration through the water column to optimize light and nutrient access while avoiding predators. However, diel vertical migration (DVM) patterns remain poorly characterized for many taxa due to limitations of labor-intensive traditional microscopy. Here, we employed high-throughput *in situ* imaging flow cytometry to investigate DVM. An Imaging FlowCytobot (IFCB) was deployed to continuously profile the vertical water column for ~10 weeks (August–October 2016) at a location in the Skagerrak, eastern North Sea. This revealed significant DVM for several morpho-taxonomic groups, including taxa belonging to ciliates, dinoflagellates, and diatoms, shifting median depth by 2–6 m between night and day. The analysis also revealed that DVM can be inferred from diel pulses in surface water biomass, which we leveraged to study DVM in an extensive IFCB time-series dataset from the central Baltic Sea (June–October in 2020 and 2021). Migratory taxa accounted for 77% and 79% of total phytoplankton biomass (size range <10–150 μm) in the Skagerrak and Baltic Sea, respectively, underscoring the ecological significance of DVM. Most populations peaked near the surface at midday, although other patterns were also observed. While many taxa displayed consistent migration behaviors across both regions, others differed—likely due to population-specific traits or local environmental conditions. Seasonal changes in migration patterns suggest a role for community turnover and shifting environmental conditions. This study highlights the prevalence of DVM in phytoplankton and showcases the power of automated, high-throughput imaging technologies to advance our understanding of plankton ecology.

## Graphical Abstract

**Graphical Abstract:**
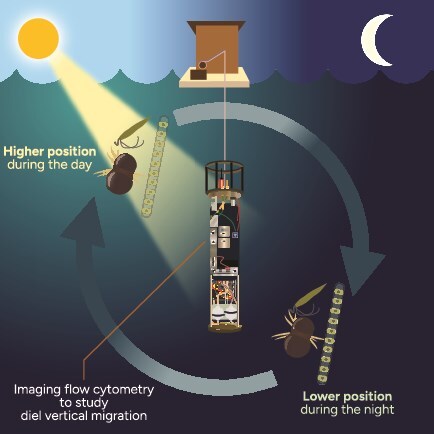

## Introduction

Although phytoplankton are traditionally considered passive drifters, many species can actively move through the water column. Roughly two-thirds of phytoplankton taxa are motile [1], either through active swimming or by regulating buoyancy. Flagellated phytoplankton, such as dinoflagellates, swim with one or more flagella [2, 3] while ciliates use numerous shorter cilia. Although ciliates are primarily heterotrophic, some species—like *Mesodinium rubrum*—function as mixotrophic phytoplankton with acquired chloroplasts [4]. Some other phytoplankton, such as many diatoms and cyanobacteria, regulate buoyancy to move vertically [5–7].

Diel (or diurnal) vertical migration (DVM), a daily rhythmic movement pattern, is undertaken by many plankton species. DVM has been extensively studied among zooplankton [8, 9] but is less understood for the phytoplankton [10, 11]. Some phytoplankton, like dinoflagellates, reside closer to the surface during the daylight period and in deeper waters during nighttime [12–15]. For example, in a study of three dinoflagellates, a ciliate, and a euglenoid in a well-stratified estuary, all five were found to migrate upwards at dawn and exhibit active movement in both directions [16]. By positioning themselves actively in the water column, phytoplankton can escape predation from zooplankton [10, 12, 17] and optimize resource acquisition [18]. In aquatic environments, there are commonly contrasting gradients of light, which is most abundant near the surface, and nutrients, often depleted in the surface waters [19]. DVM facilitates nutrient transport in the water column, and estimates suggest that vertical migration of phytoplankton supports up to 25% of total oceanic net primary production [19–21].

DVM among phytoplankton has mainly been studied in a few model species using manual optical microscopy, typically focusing on a single taxon over a short period [20–22]. In natural ecosystems, conventional optical microscopy and *in situ* chlorophyll-a (Chl-a) fluorescence measurements are the most used methods to study phytoplankton DVM patterns. Microscopy provides detailed taxonomic information, but as the analysis is time-consuming, high spatial or temporal resolution of communities is rarely achieved. Chl-a fluorescence measurements, on the other hand, provide no taxonomic differentiation but allow high spatial and temporal coverage of a system, though diel studies are hampered by nonphotochemical fluorescence quenching during daytime.

Automated imaging systems, combined with supervised machine learning-based image recognition, can bridge the gap between the conventional methods of microscopy and Chl-a fluorescence measurements, enabling long-term, high temporal resolution [23]. With automated sampling and image annotations, high-throughput surveys become feasible. Automated taxonomic classification enables identification down to genus or even species level [24, 25].

To date, few studies have applied automated imaging to investigate DVM in phytoplankton [26]. These studies typically focus on bloom events or a limited number of phytoplankton species [20, 21, 27–30]. For instance, during a *Lingulaulax polyedra* bloom, biomass accumulation was linked to nitrate depletion in deeper water layers [28]. That study employed the Imaging FlowCytobot (IFCB), a submersible, autonomous imaging flow cytometer designed to capture phytoplankton in the <10–150 μm size range [31]. This finding demonstrates how vertical migration studies with the IFCB can bring deeper knowledge about the ecology of a marine system.

In this study, we employed a submersed, vertically traversing IFCB to explore the daily vertical movements of the most common and dominant phytoplankton taxa in the Skagerrak, eastern Greater North Sea, over a 10-week period in early autumn. Our goal was to quantify the extent of DVM in the phytoplankton community and determine if certain taxa are more prone to these movements. To further investigate the extent of vertical migration, we explore an extensive surface-layer IFCB time series from a study site in the Baltic Sea, which also allowed us to compare DVM behavior between the two sites.

## Materials and Methods

### Phytoplankton sampling with Imaging FlowCytobots

**Figure 1:**
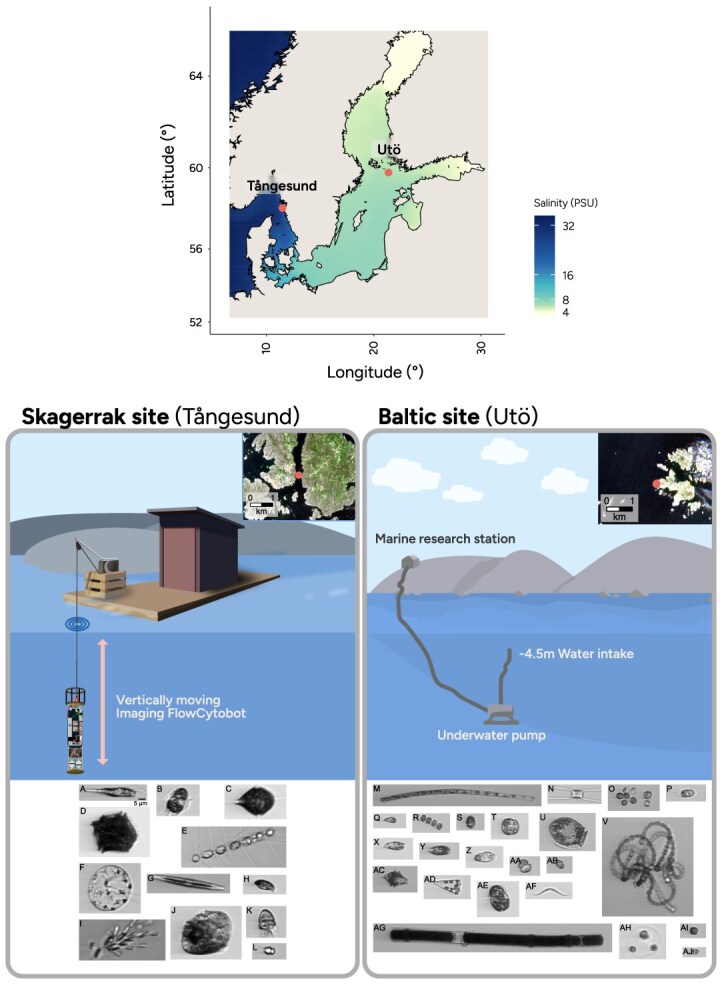
Experimental setup at the Skagerrak (top right) and Baltic (bottom) sites. The Skagerrak site overview includes a schematic of the IFCB winch setup and example images: (A) euglenophyte *Eutreptiella* spp.; (B) ciliate *M. rubrum*; (C) dinoflagellate *Scrippsiella* group; (D) dinoflagellate *Lingulaulax polyedra*; (E) diatom *Chaetoceros* spp.; (F) diatom *Thalassiosira* spp.; (G) diatom *Pseudo-nitzschia* spp.; (H) cryptophyte Cryptomonadales; (I) chlorophyte *Dinobryon* spp.; (J) dinoflagellate *Alexandrium pseudogonyaulax*; (K) ciliate *Strombidium*-like; (L) dinoflagellate *Heterocapsa rotundata*. The Baltic site set up includes an overview of the sampling (bottom left) and example images: (M) cyanobacteria *Aphanizomenon flosaquae*; (N) diatom *Chaetoceros*; (O) chlorophyte Chlorococcales; (P) cryptophyte Cryptomonadales; (Q) cryptophyte Cryptophyceae-*Teleaulax*; (R) diatom *Cyclotella*; (S) Dinophyceae obscure (contains dinoflagellates like *Heterocapsa triquetra*); (T) dinoflagellate Dinophyceae; (U) dinoflagellate *Dinophysis*; (V) *Dolichospermum-Anabaenopsis* (mainly contains *Dolichospermum*); (X) euglenophyte Euglenophyceae; (Y) euglenophyte *Eutreptiella*; (Z) dinoflagellate Gymnodiniales; (AA) dinoflagellate *Gymnodinium*-like; (AB) dinoflagellate *Heterocapsa rotundata*; (AC) dinoflagellate *H. triquetra*; (AD) diatom *Licmophora*; (AE) ciliate *M. rubrum*; (AF) chlorophyte *Monoraphidium contortum*; (AG) cyanobacteria *Nodularia spumigena*; (AH) chlorophyte *Oocystis*; (AI) stramenopile *Pseudopedinella*; (AJ) chlorophyte *Pyramimonas*. Contains modified Copernicus Sentinel-2 data, accessed via the Copernicus Data Space Ecosystem; visualization by the authors.

Phytoplankton sampling was conducted in Tångesund, Skagerrak (58.075°N, 11.493°E) (Fig. 1), adjacent to the Greater North Sea, between the 10 August and 17 October 2016. The coastal sampling site was located close to a mussel farm with a bottom depth of 20 m; solar noon was around 11:10 UTC (Coordinated Universal Time) [31]. An IFCB (McLane Research Laboratories, Inc., Falmouth, MA, USA) was operated *in situ* using a winch system mounted on a raft and cycled between 3-, 6-, 8-, 11-, 13-, and 16-m depths. One full sequence through the six depths took ~3 h. The instrument did not perform an integer number of cycles within a 24-h day, causing the sampling times to shift gradually over the study period. The IFCB analyzed ~4.7 ml of water per sample, and the camera was triggered by the detection of Chl-a fluorescence to capture images of photosynthesizing cells and avoid imaging of detritus. The sample collection in the Skagerrak was affected by technical problems during some parts of the study period, rendering the IFCB to remain at a single depth for periods of time. Unless otherwise stated, the data presented were collected when the IFCB was operating as intended at all six depths. A total of 1383 of 2829 samples were analyzed to ensure even distribution between the sample depths. NO_2_, NO_3_, NH_4_, PO_4,_ and SiO_3_ were measured weekly.

Additional sampling with another IFCB was performed in the Baltic Sea at the Utö Atmospheric and Marine Research Station (59.781°N, 21.373°E) (Fig. 1). Utö is situated on the border of the Archipelago Sea and the Northern Baltic Proper and exposed to the open sea. The device sampled seawater from an intake 250 m offshore through a tubing system, at a depth of 4.5 m (bottom depth is 20 m). The study period in the Baltic was June to October in 2020 and 2021, when the solar noon was around 10:30 UTC [31]. Both study sites are in regions considered microtidal. In the Baltic Sea, the tidal range is a few cm [32, 33], and in the Skagerrak up to 30 cm [34]. For a more detailed description of the site at Utö, see [34].

### Processing of the Imaging FlowCytobot datasets

At the Skagerrak location, expert phytoplankton taxonomists at the Swedish Hydrological and Meteorological Institute (SMHI) manually annotated IFCB images into 90 image classes (examples shown in Fig. 1) to be used as training data for machine-learning image classifiers. To ensure sufficient classifier performance, only image classes with at least 50 annotated images were included in the classifier, resulting in 42 image classes (Supplementary Fig. S1). The training dataset comprised images from the Skagerrak sampling site and research cruises in the Kattegat/Skagerrak area aboard *R/V Svea*, totaling 49 096 annotated images [35].

A convolutional neural network was trained to classify IFCB images from the Skagerrak location by using a ResNet18, pre-trained on ImageNet [36] and fine-tuned with the SMHI image library. The training followed the approach of [25], but the training data for this study were from the SMHI dataset. After training the convolutional neural network, unclassifiable images from the SYKE-plankton_IFCB_Utö_2021 dataset [25, 37] were mixed with images from the SMHI image library to determine confidence thresholds for classification. Unclassifiable images were also applied to augment the test set to better reflect natural samples. The classifier’s performance was evaluated by its precision, recall, and F1 scores, computed on the test set. The F1 score was larger than 0.80 for 28 of the 42 image classes (Supplementary Table S1).

The Skagerrak dataset was classified with the above classifier, and the biovolume and biomass of each image were computed. First, the pixel biovolume was computed according to [24, 38] (github.com/hsosik/ifcb-analysis). From the pixel biovolume, the biovolume and biomass were calculated according to equations in [39], implemented in the iRfcb R package [40]. One equation is for diatoms, which includes three classes (Bacillariohyceae, Mediophyceae, and Coscinodiscophyceae), and the other equation is for all other phytoplankton. Finally, the biomasses were converted from pg C to μg C l^−1^ by dividing by the analyzed sample volumes from the IFCB.

At the Baltic site, an established IFCB image processing pipeline was used [25] based on an updated version of the classifier. The neural network architecture and training approach, as well as the biovolume computation, are similar to those described above (see detailed description from [25]). The IFCB images from the Baltic site were classified into 106 image classes (see example images in Fig. 1). One class (diatom *Thalassiosira levanderi*) was excluded from the analysis due to poor discrimination from the diatom *Cyclotella*. The biomass of certain classes was combined with others corresponding to the same clade (e.g. image classes of *M. rubrum* from different angles). Unclassified images were included in the total biomass calculations. At the Baltic site, the classifier employs detritus classes; these classes were not included in the total biomass. After a combination of classes depicting the same clade and removal of detritus classes, 63 classes remained for the Baltic Sea site. In the Skagerrak site, detritus is negligible.

### Data processing and statistics

The images collected at different depths by the vertically cycling IFCB in the Skagerrak enable modeling of vertical distribution of biomass. The weighted mean depth (WMD) is commonly used to calculate the mean depth of a plankton population [16, 22, 41, 42]. The WMD was calculated for the total biomass concentration (including unclassified images), for each hour of the day (UTC), according to the following equation:


$$ WMD=\frac{\sum \left({A}_i{Z}_i\right)}{\sum \left({A}_i\right)} $$$$

where i is each depth sampled, A is biomass concentration (μg C l^−1^), and Z is the depth(m). By plotting the WMD over time of day, diel cycles in biomass distribution could be identified [43]. The same approach was applied to individual image classes.

To assess whether a given image class exhibited significant DVM—defined as fluctuations in WMD over the course of the day—sinusoidal curves were fitted to the WMD as a function of time. Statistical significance was evaluated through permutation testing, by randomly reassigning the time points of the observed WMD across the 24-h period. A total of 10 000 such permuted datasets were generated, and a *P*-value was calculated as the proportion of permutations in which the sinusoidal fit (*R*-value) ≥ the fit observed in the original data. To account for multiple comparisons, *P*-values were adjusted using the false discovery rate (FDR) method, applying a significance threshold at adjusted *P* < .05.

Based on the Skagerrak data, a test was developed to detect DVM from fixed-depth observations. Sinus curves were fitted to the hourly medians of near-surface (3 m) biomass, and *P*-values of the curve fits were calculated as described above. We found a cutoff *P*-value of .01 optimal for using the daily pulse as a predictor of DVM (which we had determined using vertically sampled data). The precision of the test was 0.80, the recall 0.71, and the *F*1 score 0.75 (Supplementary Fig. S2).

At the Baltic site, the IFCB was sampled at a fixed depth of 4.5 m. Here, the median biomass concentration of the total community was calculated for each hour (UTC) to assess diel fluctuations in the concentration at the inlet. Biomass concentrations were also calculated for individual image classes, and sinusoidal fits were applied to examine diel variation using the test described above.

## Results

### Distribution of phytoplankton biomass in time and space

**Figure 2:**
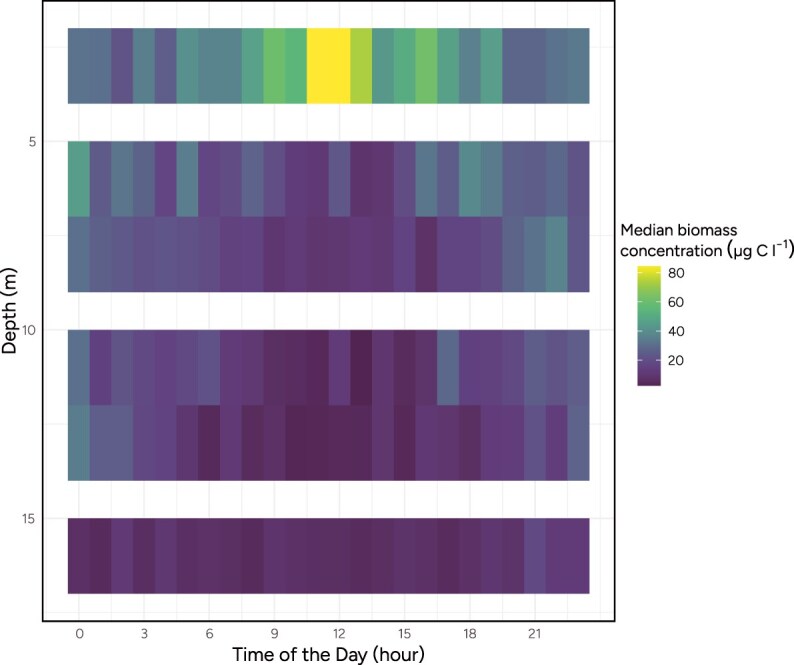
Median biomass concentration detected in the Skagerrak site by depth and hour of the day.

Skagerrak data showed clear DVM patterns of the bulk phytoplankton community (Fig. 2) averaged over the study period. At midday, the bulk phytoplankton biomass concentrates in the near-surface layer (3 m) in synchrony with a reduction of biomass in the deeper layers (6–16 m). At night, the biomass is more evenly dispersed in the water column. The DVM pattern is strongest at the 3 m depth, but visible at all depths. Importantly, although the vertical distribution of biomass fluctuated over the course of the day, the mean biomass concentration across all depths, and averaged over the study period, remained relatively constant (Supplementary Fig. S3). This suggests that most of the plankton community remained within the sampled depth or were replaced by other plankton drifting in.

**Figure 3:**
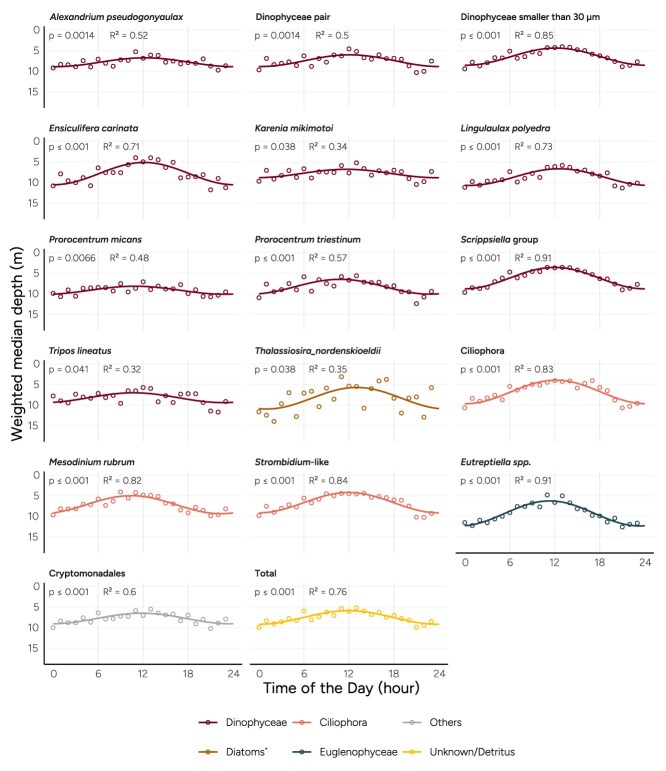
WMD of population for the different phytoplankton classes in the Skagerrak. *P*-values have been adjusted for FDR. Supplementary Figure S4 displays WMDs for nonsignificant image classes. ^*^ diatoms include the taxonomic classes Bacillariophyceae, Coscinodiscophyceae, and Mediophyceae.

The WMD of total biomass follows a cyclic diel pattern, suggesting bulk phytoplankton migration (Fig. 3). When repeating the same modeling procedure for the individual image classes, we observe that the WMD of some phytoplankton populations has a cyclic diel pattern while others maintain a stable rather fixed depth (Fig. 3, Supplementary Fig. S4, Table 1). Nearly half of the dinoflagellate image classes (7 out of 15) exhibit significant DVM (FDR-adjusted *P* < .05), typically shifting from around 10 m at night to around 5 m during the day. Of the 18 diatom image classes, only one displays significant DVM: *Thalassiosira nordenskioeldii*, but this result should be interpreted with caution as the coefficient of determination *R*^2^ is low (0.35)*.* All three groups of Ciliophora (ciliates) migrate. The largest amplitude in depth is seen for the *Eutreptiella* spp*.* (Euglenophyceae)*,* where the median depth changes from around 6 m to below 12 m in a diel cycle. When visualizing the distribution of migratory and nonmigratory classes in heatmaps (Supplementary Fig. S5), it is evident that the diel migration pattern observed in the total community (Figs 2 and 3) is primarily driven by the image classes identified as migratory.

**Table 1:** Taxonomic classes with significant DVM at the Skagerrak site and the Baltic site. For each taxonomic class, the number of image classes displaying significant DVM and the total number of image classes are displayed.

**Location**	**Skagerrak site (Tångesund)**	**Baltic Sea site (Utö)**
# Classes/Phyla with significant DVM (total)	16 (42)	25 (60)
Chlorophyceae	-	2 (3)
Chrysophyceae	0 (1)	0 (1)
Ciliophora^a^	3 (3)	3 (9)
Cyanophyceae	-	3 (10)
Diatoms^b^	1 (19)	3 (11)
Dictyochophyceae	0 (2)	1 (1)
Dinophyceae	10 (15)	7 (1)
Euglenophyceae	1 (1)	2 (2)
Others	1 (1)	4 (9)
# Samples	1303	13 482

The number of samples included in the analyses are also given. For the Skagerrak site, the number of samples includes all depths.

a Ciliophora is a phylum.

b Diatoms include the taxonomic classes Bacillariophyceae, Coscinodiscophyceae, and Mediophyceae.

### Biomass variations in surface layer: verification with Baltic Sea data

The vertical sampling setup in the Skagerrak allowed us to conclusively demonstrate DVM in several plankton taxa. Importantly, we found that taxa that displayed significant vertical migration also tended to have significant diel patterns in the surface water biomass (Supplementary Fig. S2). This makes it possible to infer DVM from surface water time-series data and allows us to expand our survey of DVM to the dataset from the Baltic Sea.

**Figure 4:**
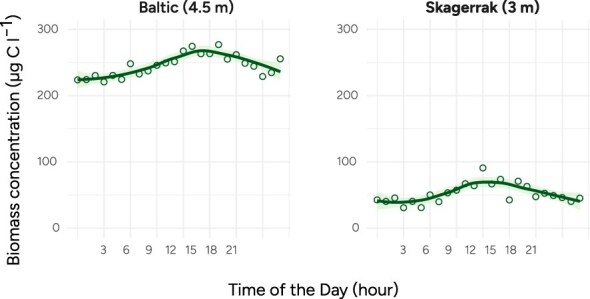
Daily variations in surface-layer total biomass concentration at the Skagerrak and Baltic Sea sites. The Baltic data covers June–October in 2020 and 2021 (13 482 samples at 4.5 m depth), and the Skagerrak data covers parts of August to October 2016 (376 samples at 3 m depth). All samples collected at 3 m in the Skagerrak were included.

**Figure 5:**
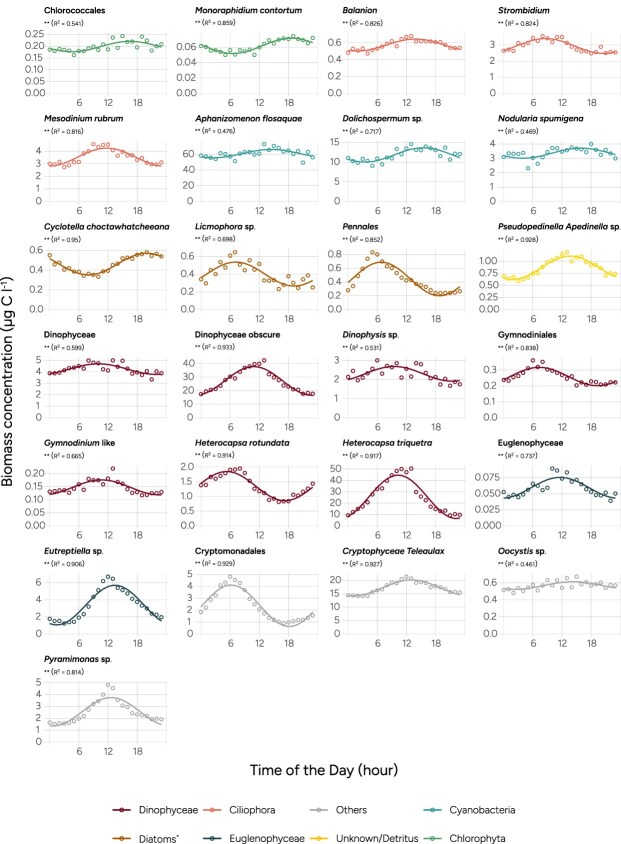
Variation in mean biomass concentration over the day for the image classes in the Baltic Sea sampling site with significant diel patterns. Sampling at 4.5 m depth.

At the Baltic Sea site, the increase in daily biomass is weaker, indicating that a smaller fraction of the total biomass migrates in diel cycles compared to at the Skagerrak site (Fig. 4). In the Skagerrak site, the midday biomass at 3 m depth was 82% higher than the midnight biomass (Fig. 4). In the Baltic Sea site, the corresponding number at 4.5 m is 21% (Fig. 4). Apart from the amplitude, the daily patterns appear similar in the two locations, with clear peaks at midday. Diel patterns can also be observed in individual image classes at the Baltic site (Fig. 5; Table 1). Of the 25 image classes with significant diel patterns, most have the peak of biomass at midday, but interestingly, there are many exceptions. For example, the different classes of filamentous Cyanobacteria (genera: *Aphanizomenon*, *Dolichospermum*, *Nodularia*) all peak later in the day compared to the rest of the phytoplankton community, around 15:00. Cryptomonadales and the dinoflagellate *Heterocapsa rotundata* both peak around 06:00.

In total, we find that 16 (38%) of the image classes in the Skagerrak, and 25 (42%) of the image classes at the Baltic Sea site display diel patterns that indicate migratory behavior (Table 1). These migratory image classes make up 77% of the classified biomass of nano and microphytoplankton in the Skagerrak site and 79% in the Baltic Sea site. The similar proportion of biomass in migratory classes at the Baltic site contrasts with the lower diel variation in surface-water biomass at the Baltic site. This may be caused by the larger variability in concentration at 3 m depth (Skagerrak site) compared to 4.5 m (Baltic site).

### Seasonal changes in biomass and diel vertical migration

**Figure 6:**
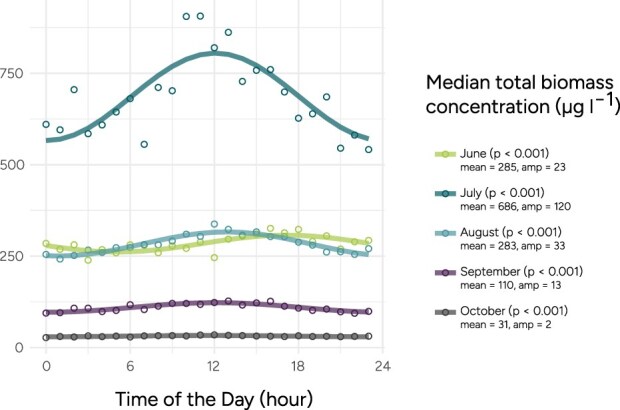
Seasonal variation in the mean biomass concentration in the Baltic Sea sampling site between June and October. Sampling at 4.5 m depth.

To investigate how vertical migration changes over the summer and autumn months, we separated the biomass observations in the Baltic Sea by month. The total observed biomass increases from June to July and then decreases from July to October (Fig. 6). All months displayed significant diel patterns, with the strongest amplitude in July. Individual image classes such as Cryptomonadales and Dinophyceae also vary in migration in the different study months (Supplementary Figs S6 and S7). This indicates that the seasonal differences in DVM are not solely explained by changing community composition, but partly by changing behaviors, although community composition did change at both sites during the study period (Supplementary Figs S8 and S9).

### Vertical nutrient profiles

Nutrient availability is a known driver for vertical migration. The concentrations of NO_2_, NO_3_, NH_4_, PO_4,_ and SiO_3_ were all positively correlated with depth at the Skagerrak site, although not significantly so for NH_4_ (Table 2). The mean concentration was 1.7 (NH_4_) to 11.3 (NO_2_) times higher at 20 m compared to 1 m. The water column at the Skagerrak site was strongly stratified, with a pycnocline at 10–15 m depth (Supplementary Fig. S10). In the end of September, the pycnocline became less pronounced. During strong wind the upper part of the water column was mixed.

**Table 2:** Mean nutrient concentration (μmol l^−1^) at different depths in the Skagerrak location.

		**NO**_**2**_	**NO**_**3**_	**NH**_**4**_	**PO**_**4**_	**SiO**_**3**_
Mean concentration of nutrient	1 m	0.03	0.20	0.50	0.09	2.21
	5 m	0.04	0.15	0.86	0.10	2.18
	10 m	0.04	0.18	0.53	0.10	2.04
	15 m	0.05	0.31	0.91	0.14	2.98
	20 m	0.39	0.57	0.82	0.28	5.79
ρ (Spearman test)		0.30^*^	0.30^*^	0.22 (ns)	0.56^***^	0.49^***^

*P*-values of Spearman rank order correlation test are indicated by symbols (ns: nonsignificant.

^^*^^: *P* ≤ .05

^***^: *P* ≤ .001).

The correlation test was conducted with depth as one vector and nutrient concentration as the other.

## Discussion

### Widespread diel vertical migration among phytoplankton

DVM is a widespread phenomenon in aquatic ecosystems yet remains challenging to quantify across diverse taxa. By leveraging automated *in situ* imaging in two contrasting marine environments, this study reveals widespread DVM behavior in phytoplankton communities. For many populations, median depth is better described by a sinusoidal model than a flat mean, indicating consistent diel movement. The vertically profiling IFCB in the Skagerrak provided high-resolution insights into this behavior. It also showed that DVM could be inferred from surface time-series data alone, allowing us to study DVM in a dataset from the Baltic Sea. Migrating taxa accounted for 77% of the biomass in classified images in the Skagerrak and 79% in the Baltic Sea, underscoring the ecological relevance of this behavior.

### Diel vertical migration patterns are largely conserved between sites

Although the plankton communities differ substantially between the two locations, some taxa occur at both, allowing for a direct comparison of DVM patterns. Five clades consistently exhibited DVM at both sites: *Eutreptiella*, *Dinophyceae*, *Cryptomonadales*, *M. rubrum and Strombidium*. All have been previously reported to exhibit DVM [44–46]. *Eutreptiella*, Dinophyceae, *Strombidium,* and *M. rubrum* followed the common reverse diel migration pattern—ascending toward the surface during the day and descending at night. However, the *Cryptomonadales* in the Baltic site peaked around 06:00, deviating from the typical reverse DVM behavior. One shared taxon—*Dinobryon*—did not exhibit DVM at either site, which is consistent with prior studies of this genus [47].

Some taxa presented contrasting patterns between sites, which may be related to differences in species-level composition or image class definitions. For instance, within the dinoflagellate genus *Prorocentrum*, we observed DVM in both species in the Skagerrak (*Prorocentrum triestinum* and *P. micans*), but not in *P. cordatum* in the Baltic Sea. From the literature, *P. micans* and *P. triestinum* are known migrators [47] whereas the behaviors of *P. cordatum* remains to be studied. The dinoflagellates *Heterocapsa rotundata* and Gymnodiniales were seen to migrate at the Baltic site but not in Skagerrak, which might be a consequence of their low abundances at the Skagerrak site, thus with insufficient statistical power to detect DVM. (Supplementary Table S2). Additionally, Gymnodiniales is a broad image-based category that may encompass different species at the two sites. When considering all taxa present at both sites, the taxonomic consistency in the diel patterns supports the interpretation that the diel patterns biomass and species composition in the Baltic are due to DVM.

### Drivers of diel vertical migration

Multiple environmental and ecological factors are known to drive DVM in phytoplankton, with incident irradiance [47], nutrient availability [48], and predation pressure [49] being the most influential. In our study, we observed a seasonal decline in DVM at the Baltic site, with migration patterns becoming less pronounced from summer to autumn. This trend aligns with shifting environmental conditions; nutrient availability generally increases while the sunlight period and intensity decrease between July and October, potentially reducing the need for active migration to balance resource acquisition. In autumn, winds are typically stronger in the Baltic location, and the surface mixed layer gets deeper and more homogeneous, hampering the ability of phytoplankton to stay at their desired vertical position. Eventually, the annual thermocline breaks down in late August–late September, and the water column is mixed deeper [40]. While significant nutrient gradients (NO₂, NO₃, PO₄, SiO₃) were observed in the Skagerrak (Table 2), limited data coverage restricts our ability to evaluate how these gradients might influence DVM over time.

Phytoplankton can adjust DVM in response to changing light conditions, e.g. to balance photosynthetic gain with protection from photoinhibition [34]. In this study, most phytoplankton biomass remained near the surface at midday, suggesting that, for many taxa present, the benefits of sustained light exposure during the day outweighed potential light stress at the measured depths. Moreover, light levels at the Baltic site at 4.5 m were likely below the level of light saturation of photosynthesis [49].

Grazing pressure is another well-documented driver of DVM in phytoplankton. For instance, in a mesocosm experiment, the dinoflagellate *Akashiwo sanguinea* exhibited increased DVM amplitude following the addition of copepod predators under a simulated day/night light cycle [50]. This behavior is thought to reduce the risk of predation, as mesozooplankton typically undertake nocturnal DVM—ascending to surface waters at night and descending during the day [41]. Mesozooplankton are believed to descend during the day to avoid being predated by fish in the sunlit upper layers. Although some zooplankton were captured by the IFCB in this study, its target detection range (<10–150 μm) [49] limits quantitative assessment of larger mesozooplankton species and their migration.

Prey availability may also shape the DVM of mixotrophic and heterotrophic taxa, along with the aforementioned drivers. In this study, many of the migrating taxa are mixotrophic. Two mixotrophic species in the Skagerrak location—*M. rubrum* and *Alexandrium pseudogonyaulax*—showed distribution patterns consistent with prey tracking. Both species were found deeper at night than during the day, suggesting that they may migrate downward to access migrating prey. A prey of *M. rubrum*, the cryptomonad image class (corresponding to the order Cryptomonadales in the class Cryptophyceae), exhibited DVM. However, the dinoflagellate *Heterocapsa rotundata*, a potential prey of *A. pseudogonyaulax*, was not found to exhibit DVM in the Skagerrak location.

### Contrasting diel vertical migration patterns in harmful microalgae

Harmful microalgae are of particular concern for shellfish aquaculture. Current monitoring in Sweden relies on integrated 0–10 m samples without standardized timing [51], which may yield inconsistent results for taxa that migrate below this depth. Our results show that several toxin-producing species (e.g. *A. pseudogonyaulax*, *L. polyedra*) actively migrate, while others (e.g. *Pseudo-nitzschia*, *Dinophysis acuminata*) do not. These findings suggest that sampling time should be standardized to ensure reliable monitoring.

In the Baltic Sea, we inferred DVM in the cyanobacterial image classes *Aphanizomenon flos-aquae*, *Nodularia spumigena*, and *Dolichospermum/Anabaenopsis* (consists of two morphologically similar genera). *A. flos-aquae* has been observed to migrate in the Baltic Sea [50], although with small fluctuations in WMD [51]. Highly buoyant cyanobacteria colonies have been collected, with floating speeds reaching 22 and 36 m per day for *A. flos-aquae* and *N. spumigena*, respectively [52]. In a previous study in the Baltic Sea, *N. spumigena* fragments were more commonly found at greater depths at night, although only two nighttime measurements were compared to one daytime measurement [53].

### Evaluating alternative explanations for diel patterns

In addition to vertical migration, other daily processes could confound DVM patterns, including periodic grazing, growth, and cell division, all of which affect abundances detected by the IFCB. As mentioned above, mesozooplankton are known to exhibit nocturnal DVM, ascending at night to feed and descending during the day to avoid predators. A meta-analysis found that mesozooplankton grazing was on average 22.6% (mode 6%) of daily primary production [52]. However, the diel variation we observed was stronger: at the Baltic site, some taxa (e.g. *Eutreptiella* sp.) showed a four-fold higher abundance at midday than at midnight. If this were solely due to asynchronous grazing and growth, it would imply unrealistically fast cell cycles with multiple divisions per day. In reality, *Eutreptiella gymnastica* grows at ~1 division d^−1^ in the Baltic Sea [53], while bulk community growth rates are 0.1–0.7 d^−1^ and grazing rates 0.05–0.3 d^−1^ [54].

Furthermore, if the daily patterns were only driven by alternating grazing and growth, we would expect variation in population biomasses throughout the day. In contrast, the observed biomass concentration averaged across depths in the Skagerrak site remains fairly constant throughout the day for most image classes. This indicates that the varying detection at fixed depths is unlikely due to changes in the total biomass of the water column, but more likely results from movement (see Supplementary Fig. S3).

The setup of the vertically profiling IFCB does not allow for precise tracking of short-term movements, making it difficult to directly measure swimming speeds. However, we can estimate average vertical velocities based on the amplitudes of the fitted depth curves from the Skagerrak location (Fig. 3). These amplitudes—defined as the vertical distance between daytime and nighttime depth extremes—ranged from 4 to 6 m across taxa. Assuming continuous upward movement during the first half of the diel cycle and downward movement during the second half, these amplitudes correspond to average swimming speeds between 0.33 and 0.5 m h^−1^. These values fall on the lower end of previous observations; for instance, one study reported upward swimming speeds of 1–2 m h^−1^ and downward speeds of 0.33–4 m h^−1^ [54].

Together, these points suggest that the observed diel changes are more likely to reflect true vertical movements than confounding ecological rhythms, but this needs to be verified with adjacent methods by estimating growth and grazing rates. In particular, the DVM of diatoms is unexpected and should be studied further with respect to stratification parameters, although buoyancy changes and mechanisms have previously been described [7, 55–57].

In summary, the phytoplankton community exhibits pronounced DVM, with substantial variation across taxa and over the season. A significant proportion of the biomass belongs to image classes that undergo vertical migration—77% and 79% in the Skagerrak and the Baltic Sea, respectively. These findings underscore the dynamic and taxon-specific nature of vertical movement within phytoplankton communities. High-throughput imaging across depth layers emerges as a powerful approach to resolve these fine-scale spatial and temporal dynamics, offering new opportunities to better understand phytoplankton ecology in marine systems.

## Supplementary Material

Supplementary_Figures_and_Tables_v3_ycag049

## Data Availability

Training data for the Skagerrak classifier are available in [35], while versions of the Baltic training data are available in [55, 58, 59]. The observational data are available at Zenodo (10.5281/zenodo.18378122).

## Code availability statement

The code used to train the image classifiers is available at https://github.com/karingare/model_construction and https://github.com/sykefi/syke-pic for the Skagerrak and Baltic datasets, respectively. The code for the analysis of DVM is available at https://github.com/karingare/diel_vertical_migration.
