# Supplementary material for: High throughput in situ imaging reveals widely occurring diel vertical migration among phytoplankton
Source: ISME Commun. 2026 Mar 9;6(1):ycag049. doi: 10.1093/ismeco/ycag049 (PMC13064659; doi:10.1093/ismeco/ycag049)
Supplement: Supplementary_Figures_and_Tables_v3_ycag049 [file supplementary_figures_and_tables_v3_ycag049.pdf]

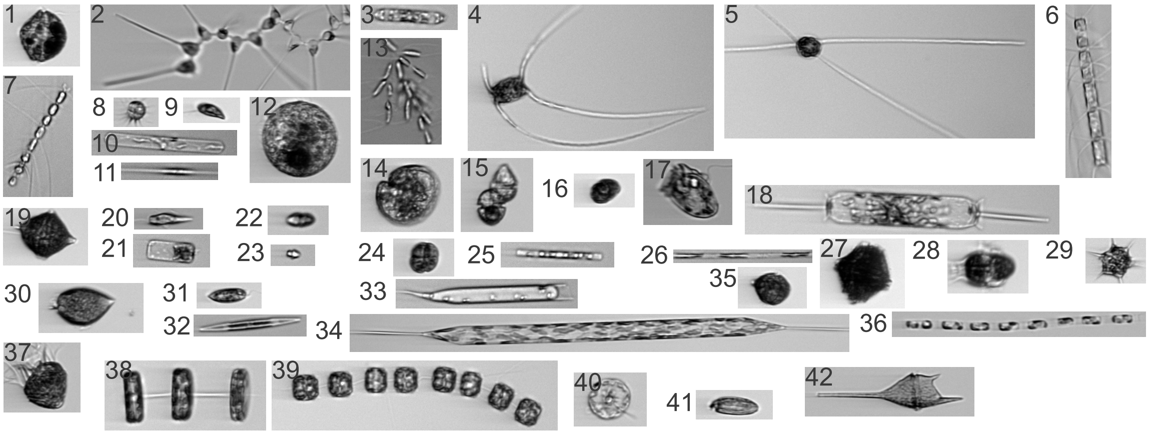

Supplementary Figure S1. Image classes in the Tångesund dataset. 1. *Alexandrium pseudogonyaulax*, 2. *Asterionellopsis glacialis*, 3. *Cerataulina pelagica*, 4. *Chaetoceros cf convolutus*, 5. *Chaetoceros danicus*, 6. *Chaetoceros spp*, 7. *Chaetoceros spp* chain, 8. Ciliophora, 9. Cryptomonadales, 10. *Cylindrotheca closterium Nitzschia longissima*, 11. *Dactyliosolen fragilissimus*, 12. Dictyochales, 13. *Dinobryon spp*, 14. Dinophyceae larger than 30, 15. Dinophyceae pair, 16. Dinophyceae smaller than 30, 17. *Dinophysis acuminata*, 18. *Ditylum brightwellii*, 19. *Ensiculifera carinata*, 20. Eutreptiella spp, 21. *Guinardia delicatula*, 22. Gymnodiniales smaller than 30, 23. *Heterocapsa rotundata*, 24. *Karenia mikimotoi*, 25. *Leptocylindrus danicus*, 26. *Leptocylindrus danicus Leptocylindrus minimus*, 27. *Lingulodinium polyedra*, 28. *Mesodinium rubrum*, 29. *Octactis speculum*, 30. *Prorocentrum micans*, 31. *Prorocentrum triestinum*, 32. *Pseudo-nitzschia spp*, 33. *Pseudosolenia calcar-avis*, 34. *Rhizosolenia Pseudosolenia*, 35. *Scrippsiella* group, 36. *Skeletonema marinoi*, 37. *Strombidium*-like, 38. *Thalassiosira gravida*, 39. *Thalassiosira nordenskioeldii*, 40. *Thalassiosira spp*, 41. *Torodinium robustum*, 42. *Tripos lineatus*.

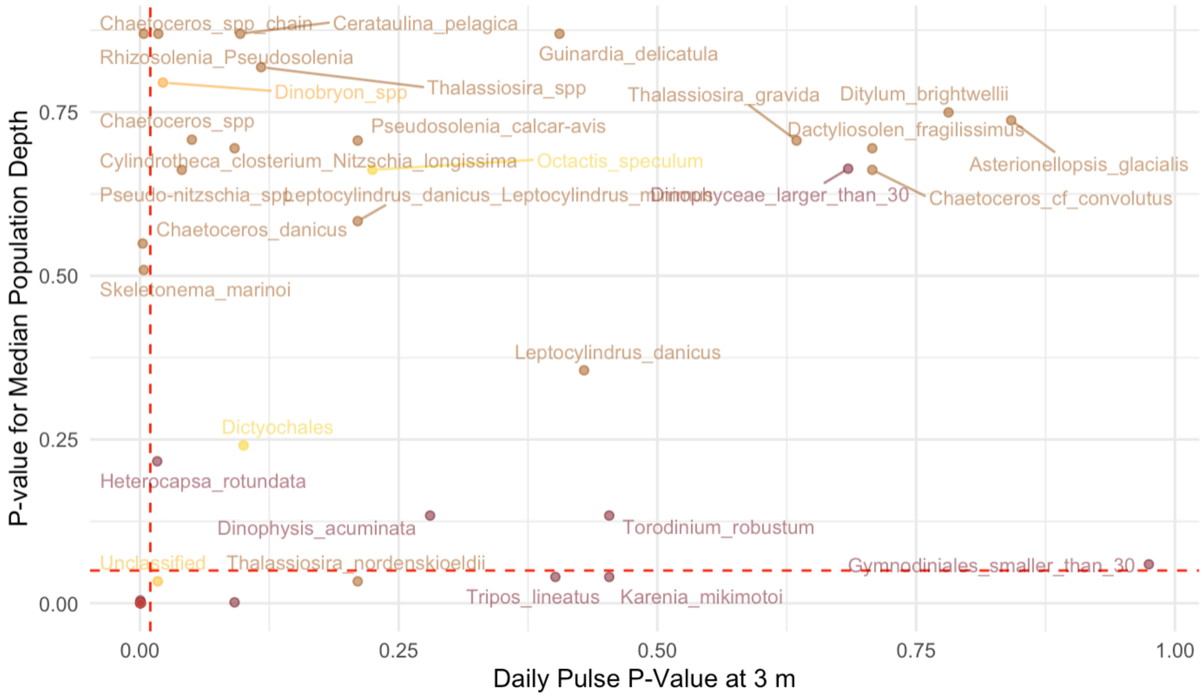

Supplementary figure S2. Relationship between significance of median population depth changes being significant and the daily pulse at 3 m sampling depth being significant for the taxa detected in the Tångesund location. Brown: diatoms, Purple: Dinophyceae, Red: Ciliates, Grey: Others, Yellow: unknown, Blue: Euglenophyceae. In the bottom left corner, the following datapoints are overlapping: Ciliophora, Cryptomonadales, Dinophyceae_pair, Dinophyceae_smaller_than_30, Ensiculifera_carinata, Eutreptiella_spp, Lingulodinium_polyedra, Mesodinium_rubrum, Prorocentrum_micans, Prorocentrum_triestinum, Scrippsiella_group, Strombidium-like.

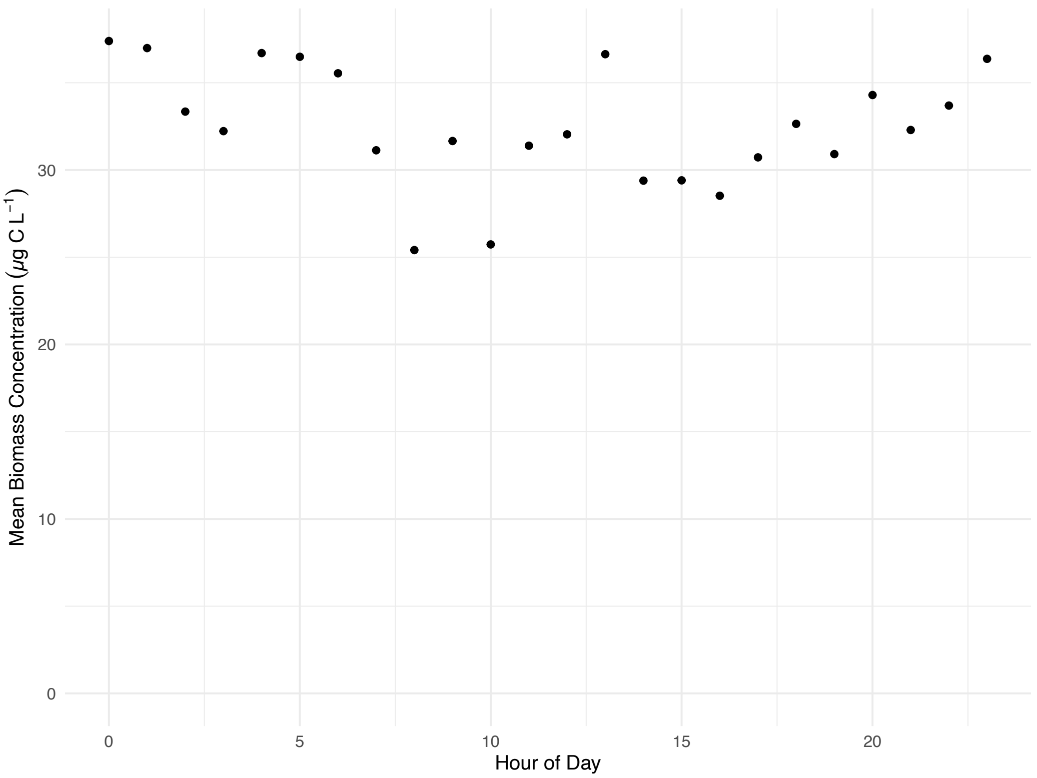

Supplementary Figure S3. The mean biomass concentrations by hour of the day in the Skagerrak site. The values are calculated by computing the mean biomass of all samples collected at a specific hour of the day.

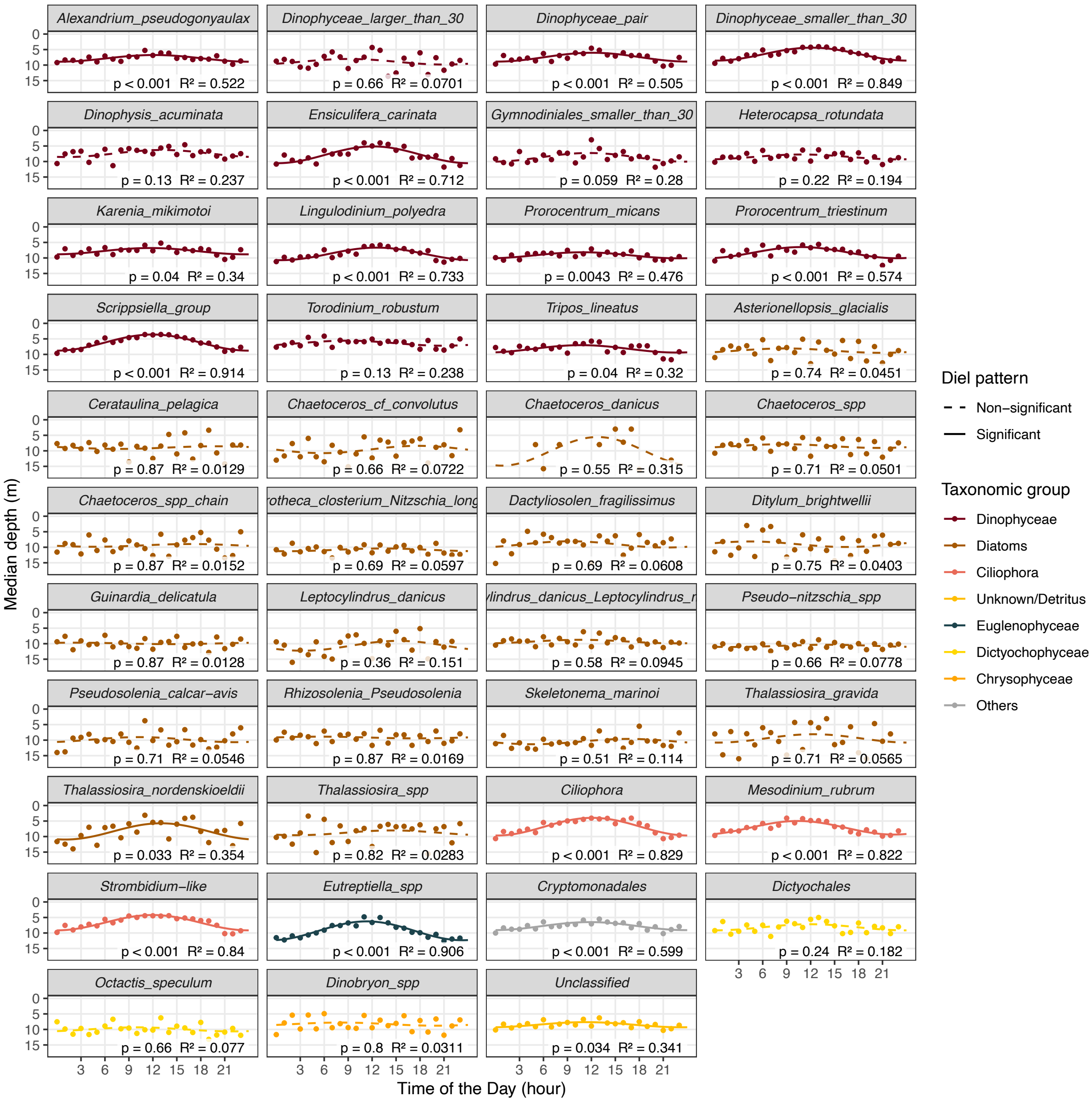

Supplementary Figure S4. Extended version of Figure 3.

Migratory classes

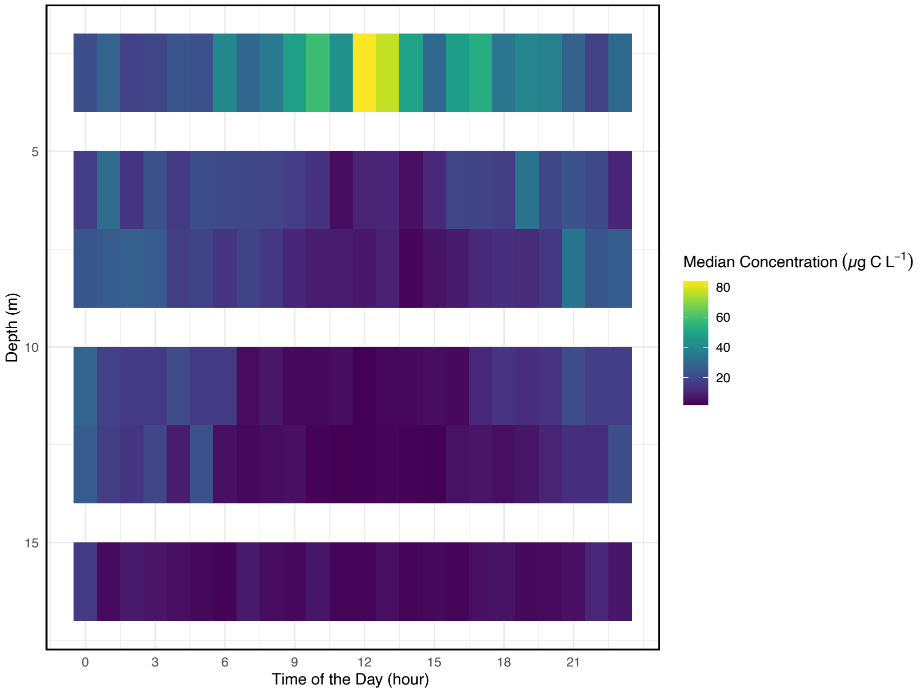

Non-migratory classes

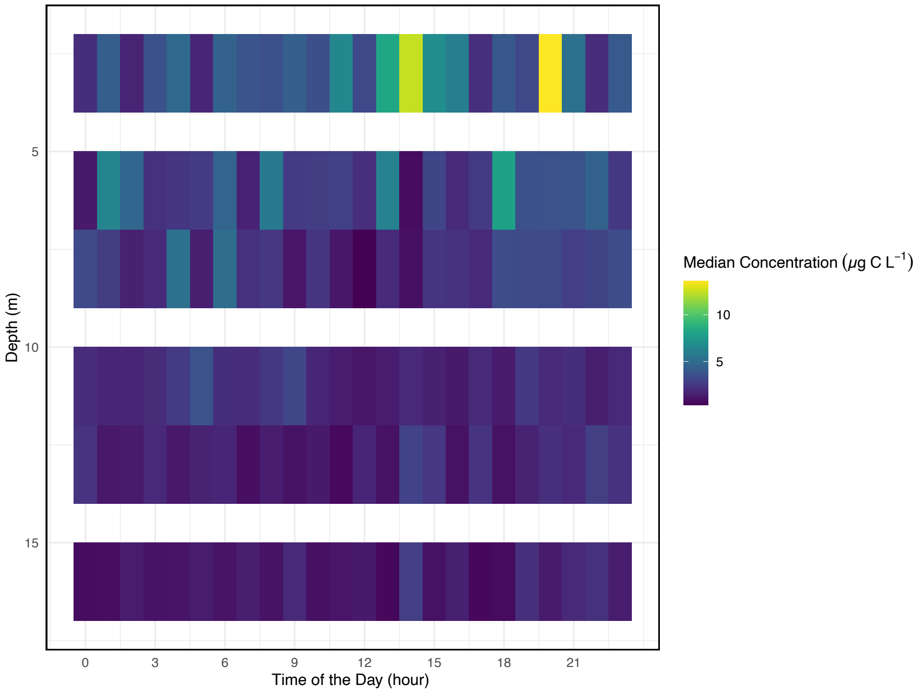

Supplementary Figure S5. Median biomass concentration detected in the Skagerrak site by depth and hour of the day, based on migratory classes and non-migratory classes, respectively. Migratory classes: Alexandrium_pseudogonyaulax, Ciliophora, Cryptomonadales, Dinophyceae_pair, Dinophyceae_smaller_than_30,

Ensiculifera_carinata, Eutreptiella_spp, Lingulodinium_polyedra, Mesodinium_rubrum, Prorocentrum_micans, Prorocentrum_triestinum, Scrippsiella_group. Strombidium-like, Thalassiosira_nordenskioeldii.

Non-migratory classes: Asterionellopsis_glacialis, Cerataulina_pelagica, Chaetoceros_cf_convolutus, Chaetoceros_danicus, Chaetoceros_spp, Chaetoceros_spp_chain, Cylindrotheca_closterium_Nitzschia_longissima, Dactyliosolen_fragilissimus, Dictyochales, Dinobryon_spp, Dinophyceae_larger_than_30, Dinophysis_acuminata, Ditylum_brightwellii, Guinardia_delicatula, Gymnodiniales_smaller_than_30, Heterocapsa_rotundata, Karenia_mikimotoi, Leptocylindrus_danicus, Leptocylindrus_danicus_Leptocylindrus_minimus, Octactis_speculum, Pseudo-nitzschia_spp, Pseudosolenia_calcar-avis, Rhizosolenia_Pseudosolenia, Skeletonema_marinoi, Thalassiosira_gravida, Thalassiosira_spp, Torodinium_robustum, Tripos_lineatus

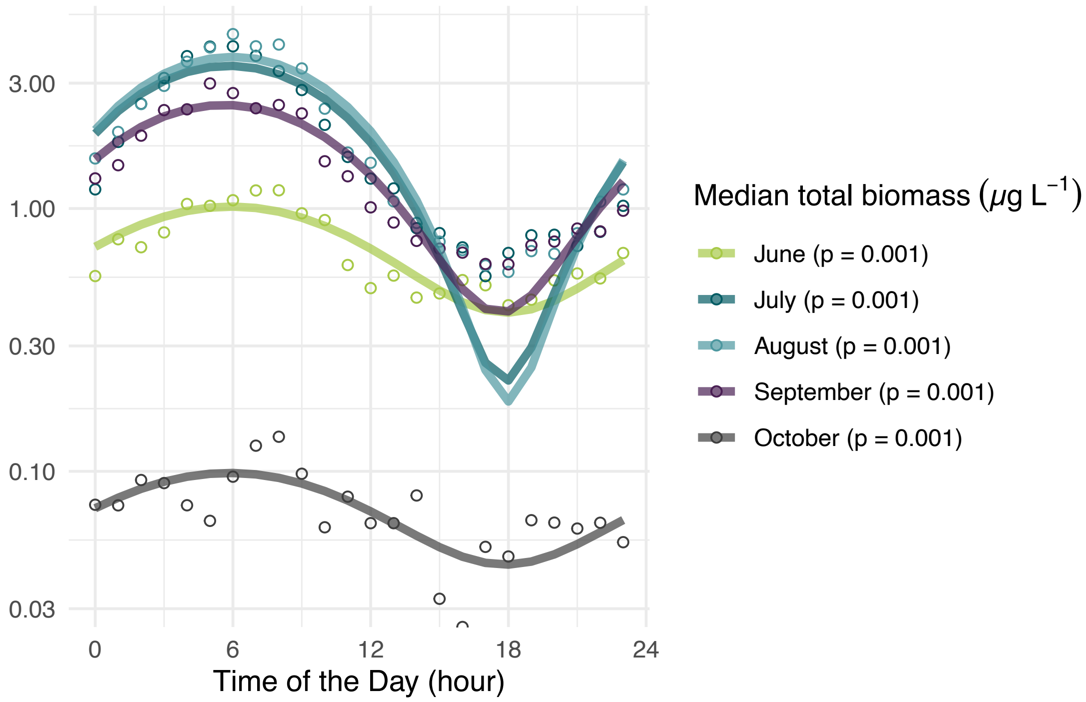

Supplementary Figure S6. Daily biomass variation for Cryptomonadales during different months at the Baltic site.

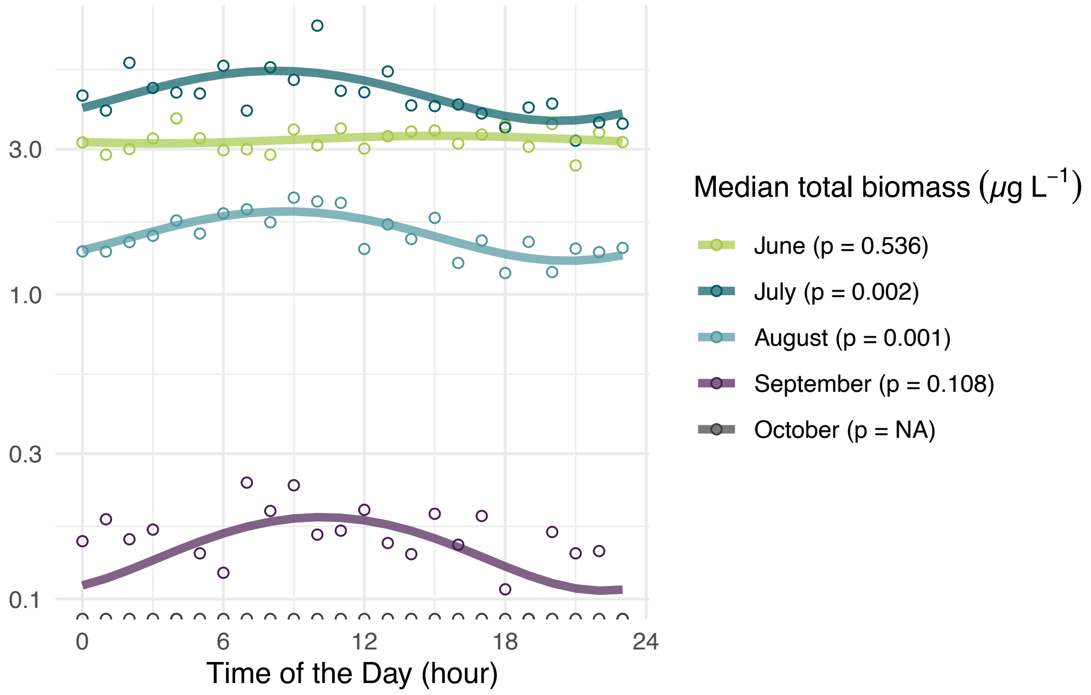

Supplementary Figure S7. Daily biomass variation for Dinophyceae during different months at the Baltic site.

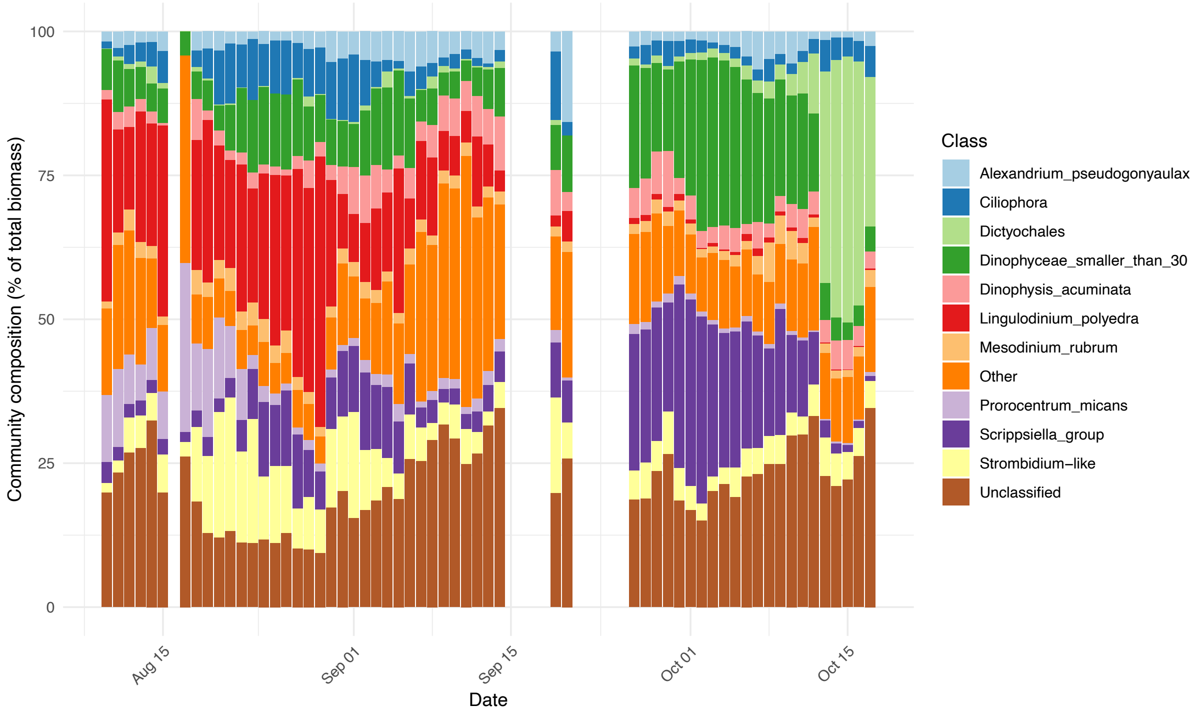

Supplementary Figure S8. Community composition at the Skagerrak site. Based on all 2,829 samples.

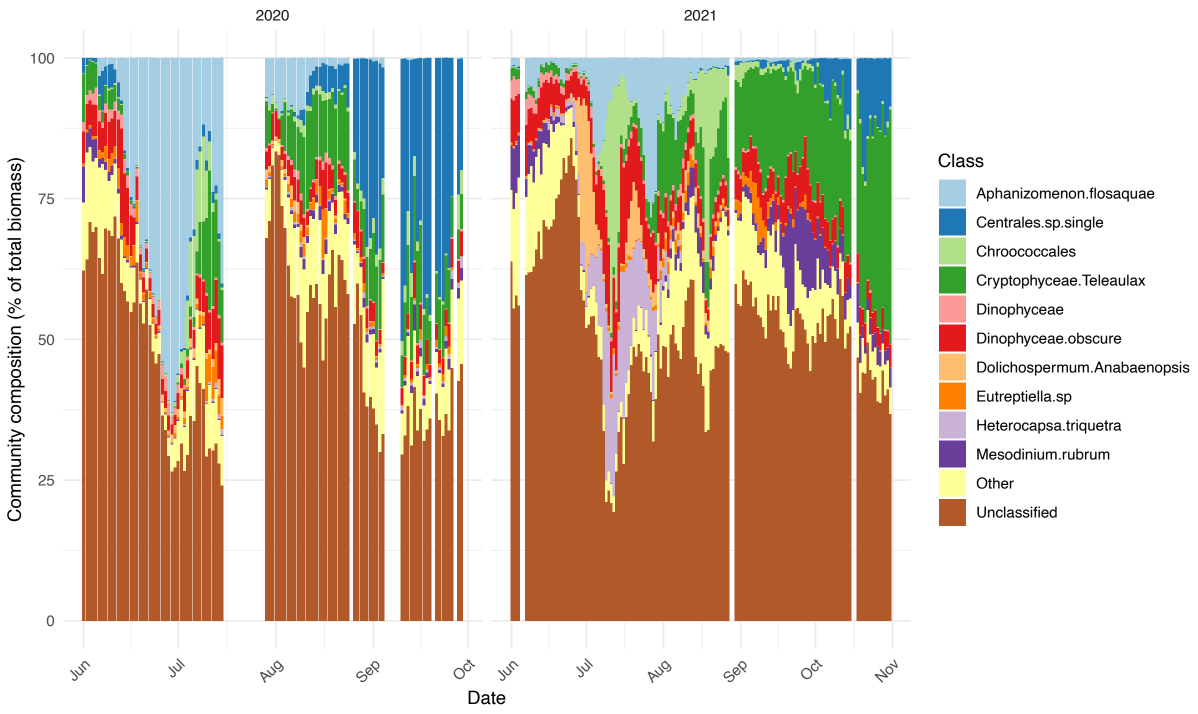

Supplementary Figure S9. Community composition at the Baltic site.

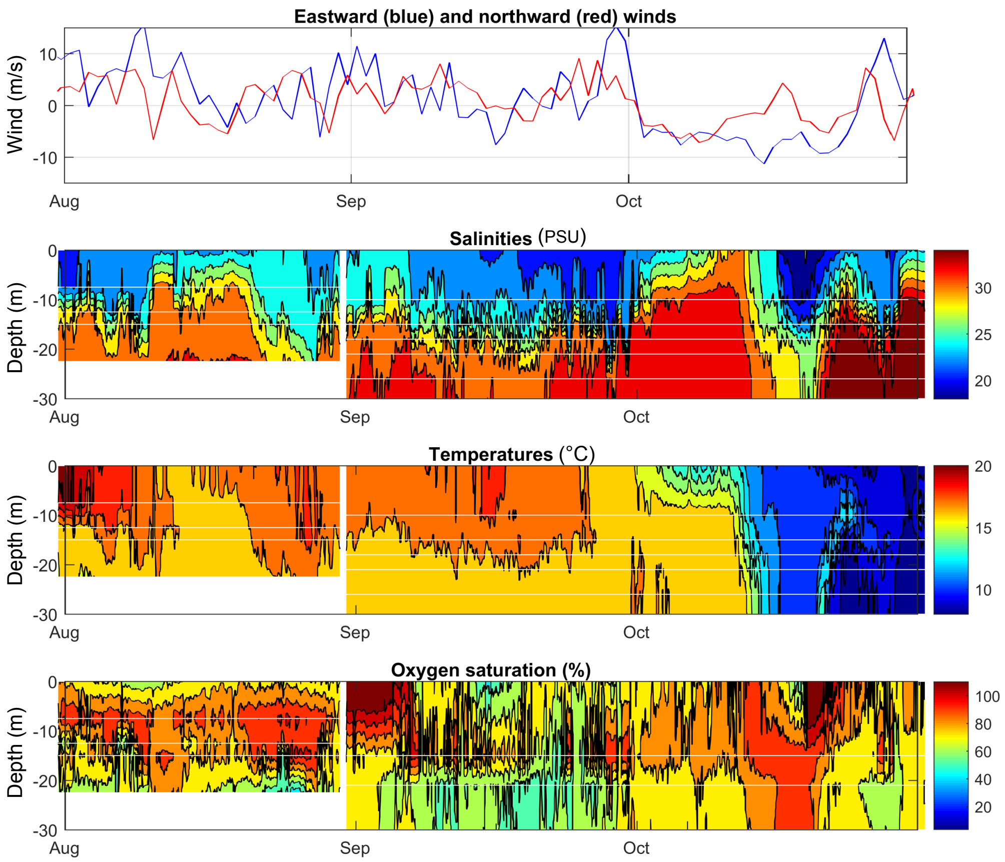

Supplementary Figure S10. Wind strengths, salinity, temperature and oxygen saturation at the Skagerrak site. Salinity is given in PSU.

Supplementary Table S1. Test set metrics for the Tångesund classifier.

Class	Precision	Recall	F1
Alexandrium_pseudogonyaulax	0.43	0.63	0.51
Asterionellopsis_glacialis	0.97	0.96	0.97
Cerataulina_pelagica	0.89	0.42	0.57
Chaetoceros_cf_convolutus	1	0.87	0.93
Chaetoceros_danicus	0.83	0.83	0.83
Chaetoceros_spp	0.99	0.82	0.9
Chaetoceros_spp_chain	0.64	0.47	0.54
Ciliophora	0.59	0.68	0.63
Cryptomonadales	0.98	0.91	0.94
Cylindrotheca_closterium_Nitzschia_longissima	1	1	1
Dactyliosolen_fragilissimus	0.95	0.87	0.91
Dictyochales	0.99	0.96	0.97
Dinobryon_spp	1	0.85	0.92
Dinophyceae_larger_than_30	0.67	0.55	0.6
Dinophyceae_pair	0.79	0.58	0.67
Dinophyceae_smaller_than_30	0.85	0.91	0.88
Dinophysis_acuminata	0.95	0.95	0.95
Ditylum_brightwellii	0.93	0.88	0.9
Ensiculifera_carinata	0.8	0.75	0.77
Eutreptiella_spp	0.96	0.81	0.88
Guinardia_delicatula	0.97	0.89	0.93
Gymnodiniales_smaller_than_30	0.57	0.75	0.65
Heterocapsa_rotundata	1	0.76	0.86
Karenia_mikimotoi	0.67	0.4	0.5
Leptocylindrus_danicus	0.94	0.52	0.67
Leptocylindrus_danicus_Leptocylindrus_minimus	0.6	0.86	0.7
Lingulaulax_polyedra	1	0.98	0.99
Mesodinium_rubrum	0.89	0.96	0.92
Octactis_speculum	1	0.93	0.96
Prorocentrum_micans	1	0.9	0.94
Prorocentrum_triestinum	0.98	0.96	0.97
Pseudo-nitzschia_spp	0.99	0.96	0.98
Pseudosolenia_calcar-avis	1	0.79	0.88
Rhizosolenia_Pseudosolenia	0.97	0.88	0.92
Scrippsiella_group	0.95	0.86	0.91
Skeletonema_marinoi	1	0.77	0.87
Strombidium-like	0.94	0.83	0.88
Thalassiosira_gravida	0.9	0.41	0.56
Thalassiosira_nordenskioeldii	0.25	0.33	0.29
Thalassiosira_spp	0.56	0.26	0.36
Torodinium_robustum	1	0.82	0.9
Tripos_lineatus	0.97	1	0.99

Supplementary table S2. Observed biomass in the Skagerrak site.

Class	# images	# samples	Total biomass (µg C)
Alexandrium_pseudogonyaulax	4097	1099	6.44
Asterionellopsis_glacialis	8336	781	4.94
Cerataulina_pelagica	697	426	0.26
Chaetoceros_cf_convolutus	110	96	0.01
Chaetoceros_danicus	12	12	0
Chaetoceros_spp	45537	1201	5.49
Chaetoceros_spp_chain	359	244	0.02
Ciliophora	6018	949	8.25
Cryptomonadales	101670	1377	4.18
Cylindrotheca_closterium_Nitzschia_longissima	506	374	0.01
Dactyliosolen_fragilissimus	195	170	0.18
Dictyochales	522	350	2.37
Dinobryon_spp	1238	342	0.16
Dinophyceae_larger_than_30	305	277	0.64
Dinophyceae_pair	1968	757	2.73
Dinophyceae_smaller_than_30	142162	1383	33.6
Dinophysis_acuminata	1226	712	7.68
Ditylum_brightwellii	102	92	0.09
Ensiculifera_carinata	1651	524	1.97
Eutreptiella_spp	3012	982	0.38
Guinardia_delicatula	826	505	0.15
Gymnodiniales_smaller_than_30	166	149	0.01
Heterocapsa_rotundata	6783	1137	0.19
Karenia_mikimotoi	4838	1172	1.79
Leptocylindrus_danicus	118	104	0
Leptocylindrus_danicus_Leptocylindrus_minimus	1203	577	0.06
Lingulodinium_polyedra	8268	891	19.62
Mesodinium_rubrum	17733	1365	5.69
Octactis_speculum	700	509	3.26
Prorocentrum_micans	5537	1029	5.35
Prorocentrum_triestinum	10451	1194	1.54
Pseudo-nitzschia_spp	25180	1100	0.46
Pseudosolenia_calcar-avis	184	161	0.05
Rhizosolenia_Pseudosolenia	2515	819	0.59
Scrippsiella_group	97724	1356	30.26
Skeletonema_marinoi	1976	639	0.02
Strombidium-like	20953	1113	16.55
Thalassiosira_gravida	72	68	0.12
Thalassiosira_nordenskioeldii	171	127	0.05
Thalassiosira_spp	248	205	0.1
Torodinium_robustum	569	370	0.38
Tripos_lineatus	817	504	1.35
Unclassified	347912	1383	41.42
